# Family History for Neurodegeneration in Multiple System Atrophy: Does it Indicate Susceptibility?

**DOI:** 10.1002/mds.29202

**Published:** 2022-08-27

**Authors:** Fabian Leys, Sabine Eschlböck, Nicole Campese, Philipp Mahlknecht, Marina Peball, Georg Goebel, Victoria Sidoroff, Roberta Granata, Vincenzo Bonifati, Johannes Zschocke, Stefan Kiechl, Werner Poewe, Klaus Seppi, Gregor K. Wenning, Alessandra Fanciulli

**Affiliations:** ^1^ Department of Neurology Medical University of Innsbruck Innsbruck Austria; ^2^ Neurology Unit, Department of Clinical and Experimental Medicine University of Pisa Pisa Italy; ^3^ Department of Medical Statistics, Informatics and Health Economics Medical University of Innsbruck Innsbruck Austria; ^4^ Department of Clinical Genetics University Medical Center Rotterdam The Netherlands; ^5^ Institute of Human Genetics Medical University of Innsbruck Innsbruck Austria

**Keywords:** multiple system atrophy, Parkinson's disease, genetics, family history, familial

Multiple system atrophy (MSA) is a rare, rapidly‐progressive neurodegenerative disorder, neuropathologically characterized by oligodendroglial α‐synuclein aggregates.^1^ While in Parkinson's disease (PD), a neuronal α‐synucleinopathy, both monogenic forms and a polygenic risk profile are known,^2^ MSA is generally considered a sporadic disorder.^1^ A family history (FH) for parkinsonism or other neurodegenerative disorders may in fact occur in people with MSA, but the contribution of genetic factors to MSA pathogenesis is not fully understood to date.^3^, ^4^

Here we retrospectively assessed the frequency rates of FH for parkinsonism, dementia, tremor, ataxia, or motor neuron disease within first‐to‐third‐degree relatives of people included in the Innsbruck MSA Registry (n = 144), and compared them with historical MSA cohorts (cumulative n = 1173), Innsbruck‐based PD cases (n = 226), and published population‐based controls (cumulative n = 20,784). A detailed methodological description is provided in Supplementary Document 1.

Forty‐five MSA cases (40%) had a positive FH for neurodegenerative disorders, with parkinsonism being most prevalent (n = 26, 18%). FH rates mostly matched or exceeded those of historical MSA cohorts (Fig. 1A). The cumulative first‐to‐third‐degree FH rates for neurodegenerative disorders and familial clustering (ie, ≥2 affected relatives) remained comparable between the MSA and PD cohort (Fig. 1B). Compared to pooled population‐based controls, first‐degree FH rates for dementia were significantly lower in both the MSA and PD cohorts, whereas the rate of first‐degree FH for parkinsonism in MSA cases (10%, 95% CI 6–17) was between that of PD (17%, 95% CI 13–23; *P* = 0.079) and population‐based controls (6%, 95% CI 5–6; *P* = 0.012; Fig. 1C and Supplementary Document 2).

**FIG. 1:**
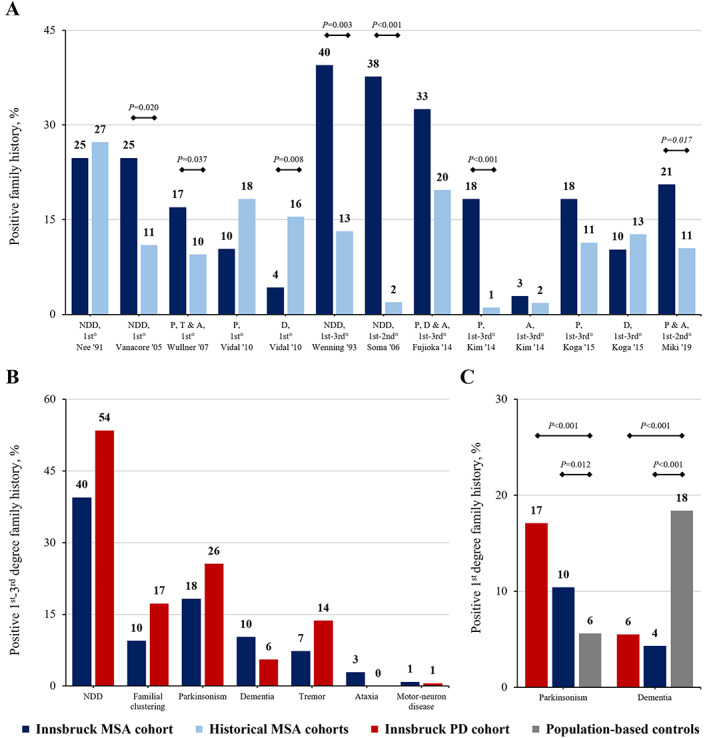
(A) Domain‐ and degree‐adjusted family history (FH) rates in the Innsbruck multiple system atrophy (MSA) versus historical MSA cohorts. (**B)** Cumulative FH rates in the Innsbruck MSA versus Parkinson's disease (PD) cohort. (**C)** FH rates for first‐degree parkinsonism and dementia in the Innsbruck MSA and PD cohorts compared to population‐based elderly controls. NDD, neurodegenerative disorders; P, parkinsonism; T, tremor; A, ataxia; D, dementia. [Color figure can be viewed at wileyonlinelibrary.com]

The ultimate mechanisms underlying MSA pathogenesis remain largely unknown.^1^, ^5^ The high frequency of FH for parkinsonism in people with MSA, close to that of PD and exceeding the one observed in population‐based elderly controls, supports the contention that multiple, yet unidentified genetic variants might contribute to MSA pathogenesis. It also suggests a shared genetic susceptibility to the development of MSA and PD.

Our study has limitations. FH history was collected retrospectively, carrying the risk for a documentation bias, and with the *FH method*, which obtains information on FH exclusively from patients and may both under‐ and overestimate FH rates.^6^ In the age of genomic medicine, however, FH still represents a valuable tool to assess the heritability of a given disorder, especially if genetic methods fail to disclose a causal relation. Non‐neurodegenerative causes of tremor, dementia, or parkinsonism were also not systematically excluded in the relatives of our patients; genetic testing was available in a small percentage of patients only; and neuropathological confirmation in none. We also did not include an age‐ and sex‐matched control group, but compared our data with the cumulative results of historical MSA cohorts and large population‐based studies in aging individuals.

Similar to PD, genetic susceptibility variants — if discovered for MSA — may be exploited for identifying persons at risk of developing the disease or in very early stages thereof, when putative neuroprotective strategies should ideally be most effective.^7^ Understanding the genetic underpinnings of the MSA pathological cascade might ultimately point out new therapeutic targets for this currently untreatable condition.

## Financial Disclosure Related to Research Covered in this Article

Academic study without external funding. Dr Leys was supported by the Stichting ParkinsonFonds, US MSA Coalition and Dr Johannes & Hertha Tuba Foundation.

## Ethical and Regulatory Aspects

Due to its retrospective nature and initiation before July 2020, neither written informed consent nor ethic approval was required for the present study. This study was conducted in accordance with the Declaration of Helsinki and the current European Data Protection Regulation. We confirm that we have read the Journal's position on issues involved in ethical publication and affirm that this work is consistent with those guidelines. The first and last named authors take full responsibility for the integrity of the data and the accuracy of the data analysis.

## Author Roles

(1) Research project: A. Conception, B. Organization, C. Execution; (2) Statistical Analysis: A. Design, B. Execution, C. Review and Critique; (3) Manuscript: A. Writing of the First Draft, B. Review and Critique.

F.L.: 1B, 1C, 2B, 3A

S.E.: 1C, 2C, 3B

N.C.: 1C, 2C, 3B

P.M.: 1A, 1C, 2C, 3B

M.P.: 1C, 2C, 3B

G.G.: 1A, 2A, 2C, 3B

V.S.: 1A, 2C, 3B

R.G.: 1A, 2C, 3B

V.B.: 1A, 2C, 3B

J.Z.: 1A, 2C, 3B

S.K.: 1A, 2C, 3B

W.P.: 1A, 2C, 3B

K.S.: 1A, 2C, 3B

G.K.W.: 1A, 2C, 3B

A.F.: 1A, 1B, 1C, 2A, 2C, 3B

## Full Financial Disclosures of All Authors (for the Preceding 12 Months)

F.L., S.E., N.C., P.M., M.P., G.G., V.S., R.G.: none. V.B.: receives research grants from the Stichting ParkinsonFonds and from Alzheimer Nederland (The Netherlands); honoraria from the International Parkinson and Movement Disorder Society, as Chair of the Congress Scientific Program Committee 2019–2021; and from Elsevier Ltd, as Co‐Editor‐in‐Chief of *Parkinsonism & Related Disorders*, outside of the submitted work. J.Z.: none. S.K.: reports support from the Austrian Research Promotion Agency FFG, outside of the submitted work. W.P.: reports receiving personal fees from AbbVie, AFFiRiS, AstraZeneca, BIAL, Boston Scientific, Britannia, Intec, Ipsen, Lundbeck, NeuroDerm, Neurocrine, Denali Pharmaceuticals, Novartis, Orion Pharma, Prexton, Teva, UCB, and Zambon. He receives royalties from Thieme, Wiley Blackwell, Oxford University Press, and Cambridge University Press and grant support from The Michael J. Fox Foundation, EU FP7, and Horizon 2020, outside of the submitted work. K.S.: reports personal fees from Teva, UCB, Lundbeck, AOP Orphan Pharmaceuticals AG, Roche, Grünenthal, Stada, Licher Pharma, Biogen, BIAL, and Abbvie; honoraria from the International Parkinson and Movement Disorders Society; research grants from FWF Austrian Science Fund, The Michael J. Fox Foundation, and AOP Orphan Pharmaceuticals AG, outside the submitted work. G.K.W.: reports consultancy and lecture fees from AbbVie, AFFiRiS AG, AstraZeneca, Biogen, Biohaven, Inhibicase, Lundbeck, Merz, Ono, Teva, and Theravance, and research grants from the Austrian Science Fund (FWF), the Austrian National Bank, the US MSA Coalition, Parkinson Fonds Austria, the Dr Johannes und Hertha Tuba Foundation, and the International Parkinson and Movement Disorder Society, outside of the submitted work. A.F.: reports royalties from Springer Verlag, speaker fees and honoraria from Impact Medicom, Theravance Biopharma, AbbVie, the International Parkinson Disease and Movement Disorders Society, the Austrian Neurology Society, the Austrian Autonomic Society, and research grants from the Parkinson Fond, the US MSA Coalition, the Dr Johannes and Hertha Tuba Foundation, and the Austrian Exchange Program, outside of the submitted work.

## Supporting information


**Appendix S1.** Supporting Information.

## Data Availability

The data supporting the findings of this study are available upon reasonable request from any qualified investigator.
