# Supplementary material for: Family History for Neurodegeneration in Multiple System Atrophy: Does it Indicate Susceptibility?
Source: Mov Disord. 2022 Aug 27;37(11):2310–2. doi: 10.1002/mds.29202 (PMC9804463; doi:10.1002/mds.29202)
Supplement: Supplementary file 1 — Appendix S1. Supporting Information. [file MDS-37-2310-s001.zip › MDS_29202_Suppl Doc 1_Letter_Family history for neurodegeneration in MSA.docx]

**Family history for neurodegeneration in multiple system atrophy:
does it indicate susceptibility?
A clinico-genetic study based on the Innsbruck MSA Registry**

**Methodology**

**Study population**

We retrospectively screened the Innsbruck Multiple System Atrophy (MSA) Registry for patients fulfilling the second consensus criteria^1^ for probable MSA *OR* possible MSA with at least 3 years disease duration and 12 months follow-up from the baseline visit, who provided informative family history (FH) for neurodegenerative disorders within first-to-third degree relatives. For comparison, the Austrian Parkinson Registry was searched for Innsbruck patients fulfilling the Queen Square Brain Bank criteria^2^ for definite Parkinson’s disease (PD) with at least 5 years disease duration and 12 months follow-up at the final visit, who also provided informative FH. All patients were examined at the Department of Neurology, Medical University of Innsbruck, Austria, between November 1997 and April 2021.

**Data collection**

Data on FH and other clinic-demographic information was retrospectively collected from available electronic and handwritten medical records. Information concerning FH was retrieved following a uniform scheme and required an unequivocal, clear documentation of either a positive or negative FH for at least first-degree relatives in at least one out of the five investigated domains (i.e., parkinsonism, dementia, tremor, ataxia, or motor-neuron disease). Whenever a positive FH was documented, e.g., for a first-degree relative in the parkinsonism domain, but no further information was given, we assumed that the physician has thoroughly enquired about FH for neurodegenerative disorders and – subsequently – marked the other domains with “no” within first-to-third degree relatives. Whenever FH was reported to be “negative for neurodegenerative disorders*”,* we marked a fully negative FH for all investigated domains up to the third-degree. In case the documentation of a negative FH was limited to a specific domain or degree, e.g., “no history of parkinsonism within first-degree relatives”, the respective cases were considered for domain and degree specific, i.e., first-degree parkinsonism, subanalyses only. Due to the retrospective design, we were unable to systematically exclude non-neurodegenerative etiologies underlying a positive FH; however, in case of a clear documentation of, e.g., vascular dementia or drug-induced parkinsonism, FH was counted as negative. Cases with absent or insufficient (e.g., “other cases of parkinsonism in the family”) FH documentation were excluded. If, either at the discretion of the physician or the patients themselves, there were any uncertainties documented whether a relative was affected by a neurodegenerative disorder, the respective cases were excluded too. Familial clustering was defined as at least two first-to-third degree relatives suffering from a neurodegenerative disorder.

A detailed description of the clinical information retrieval strategy for demographics, outcome of an eventual genetic testing, initial clinical features, comorbidities, and the Hoehn-&-Yahr stage^3^ is provided in Supplementary Table 1.

**Literature search**

To determine the frequency rates of FH for neurodegenerative disorders in historical MSA cohorts and population-based elderly controls, we searched the PubMed database up to November 2021. The following combination of keywords was used to identify FH frequency rates in historical MSA cohorts: [“multiple system atrophy”] AND [“familial” OR “family history” OR “familial Parkinson” OR “familial parkinsonism” OR “familial dementia” OR “familial tremor” OR “familial ataxia” OR “familial motor neuron disease” OR “family history of Parkinson” OR “family history of parkinsonism” OR “family history of dementia” OR “family history of tremor” OR “family history of ataxia” OR “family history of motor neuron disease” OR “family history of neurodegenerative disorders” OR “genetic”].

The following combination of keywords was used to identify FH frequency rates in population-based controls: [“population” OR “population-based”] AND [“familial” OR “family history”] AND [“Parkinson” OR “parkinsonism” OR “dementia” OR “tremor” OR “ataxia” OR “motor neuron disease” OR “neurodegenerative disorders”].

Moreover, we performed a search including names of large (≥1,000 persons) population-based longitudinal studies mentioned by Seematter-Bagnoud *et al.*^4^ that assessed ageing, cognition, or functional and cognitive decline in both sexes above the age of 50 years, its related articles, and those of the authors’ experience with the study abbreviation, if available, or the full title: [“ADAMS” OR “AHEAD” OR “AMSTEL” OR “ATHLOS” OR “Australian Longitudinal Study of Ageing” OR “Bambui Health and Ageing Study” OR “CARLA” OR “BCRA” OR “CC75C” OR “CHANCES” OR “COSMIC” OR “CSHA” OR “ELSA” OR “EPESE” OR “EPIDOSO” OR “EVA” OR “Groningen Longitudinal Ageing Study” OR “Hong Kong old-old Survey” OR “HRS” OR “ILSA” OR “Japanese Longitudinal Studies” OR “Kungsholmen Project” OR “LASA” OR “LEILA 75+” OR “Leiden 85-plus study” OR “Longitudinal Survey of Ageing” OR “LSOA” OR “Manitoba Study of Health and Ageing” OR “Maracaibo Ageing Study” OR “MHCPS” OR “MoVIES” OR “MRC-CFAS” OR “NEDICES” OR “NORA” OR “Nottingham Longitudinal Study of Activity and Ageing” OR “Odense Study” OR “PAQUID” OR “Rotterdam Study” OR “Shanghai Survey of Dementia” OR “SHARE” OR “TamELSA” OR “TILDA” OR “TMIG-LISA” OR “WHICAP”] AND [“familial” OR “family history”] AND [“Parkinson” OR “parkinsonism” OR “dementia” OR “tremor” OR “ataxia” OR “motor neuron disease” OR “neurodegenerative disorders”].

We also hand-searched studies from the citing literature or known to the authors. To be eligible for comparison, data on FH had to deal specifically with at least one of the five abovementioned domains in first-degree relatives. Population-based samples likely affected by any of the investigated neurodegenerative disorders were excluded. Only articles published in English language were considered.

**Statistical analyses**

Qualitative variables were summarized by frequency (percentage) and quantitative variables by median [25^th^; 75^th^ percentile]. Depending on the sample size, we used the Shapiro-Wilk or Kolmogorov-Smirnov test to test for normality. Differences in quantitative variables were assessed with the Student’s *t* or Mann–Whitney *U* test, depending on data distribution. Qualitative variables were analyzed with the Pearson’s Chi-squared, Fisher’s exact, or Fisher-Freeman-Halton test, where appropriate. Confidence intervals (c.i.) were calculated according to the modified Wald method.^5^ We applied a Benjamini-Hochberg correction for multiple comparison with an unadjusted α-level of *P*<0.05.

First, we determined the FH frequency rates in the Innsbruck MSA cohort. After adjusting for the respective FH domain(s) and degree(s), e.g., first, second, or third, we compared FH frequency rates between the present and historical MSA cohorts. Next, we investigated the differences in FH frequency rates and other clinic-demographic characteristics between the MSA and PD cohorts. Finally, we pooled the available FH frequency rates for neurodegenerative disorders of population-based elderly controls identified by the literature search and compared them with both the MSA and PD cohorts.

A post-hoc sensitivity analysis was added to identify any differences in the frequency rates of positive FH between MSA patients with last available follow-up before and after publication of the second MSA consensus criteria^1^ (August 2008), which first discarded a positive FH from the exclusion criteria for a MSA diagnosis. A schematic overview of the analytic approach is provided in Supplementary Fig. 1.

The statistical analysis was performed with IBM SPSS^®^ Statistics v.27.0 (IBM Corporation, Armonk, NY, USA) and MedCalc^®^ (MedCalc Software Ltd, Ostend, Belgium, Europe). A two-tailed *P*-value of <0.05 was considered statistically significant. The data supporting the findings of this study are available upon reasonable request from any qualified investigator.

**Supplementary Table 1.** Clinic-demographic characteristics assessed in the MSA and PD cohorts

| **Variable** | **Definition** |
| --- | --- |
| Sex | Documented sex: female or male. |
| Age at onset | Age at manifestation of the initial clinical symptoms in years. |
| Age at the baseline/final visit | Age at the baseline/final visit in years. |
| Disease duration at the baseline/final visit | Time from age at onset to the baseline/final visit in months. |
| Follow-up time | Time from the baseline to final visit in months. |
| Genetic testing | Documented positive genetic testing for any variants that might cause MSA-mimicking phenotypes^6^ or inherited forms of parkinsonism.^7, 8^ |
| Initial clinical feature | Defined as the initial clinical presentation of any motor symptoms (parkinsonism or cerebellar ataxia), autonomic features (orthostatic intolerance or neurogenic bladder disturbances), or combined motor-autonomic features, if both were reported to have occurred simultaneously.^9^ We assigned the initial clinical presentation to motor symptoms when it remained unclear whether secondary factors could have been accountable for orthostatic intolerance or bladder disturbances. |
| Cardiovascular diseases | Documented diagnosis of any cardio- and/or cerebrovascular diseases at the baseline/final visit. |
| Diabetes mellitus | Documented diagnosis of diabetes mellitus at the baseline/final visit. |
| Unified MSA Rating Scale Part IV score | If available, the Unified MSA Rating Scale Part IV^10^ score at the baseline/final visit was adopted from medical records or, otherwise, post-hoc assessed based on the neurological examination and history. We did not assess the Unified MSA Rating Scale Part IV score in patients not fulfilling MSA diagnostic criteria^1^ at the baseline visit. |
| Hoehn-&-Yahr stage | If available, the Hoehn-&-Yahr stage at the baseline/final visit was adopted from medical records or, otherwise, post-hoc assessed according to the modified Hoehn-&-Yahr scale^3^ based on the neurological examination and history. Presence of at least one feature suggesting parkinsonism^1, 2^ was required to assess the Hoehn-&-Yahr stage. |

**Supplementary Fig. 1** Analytic approach


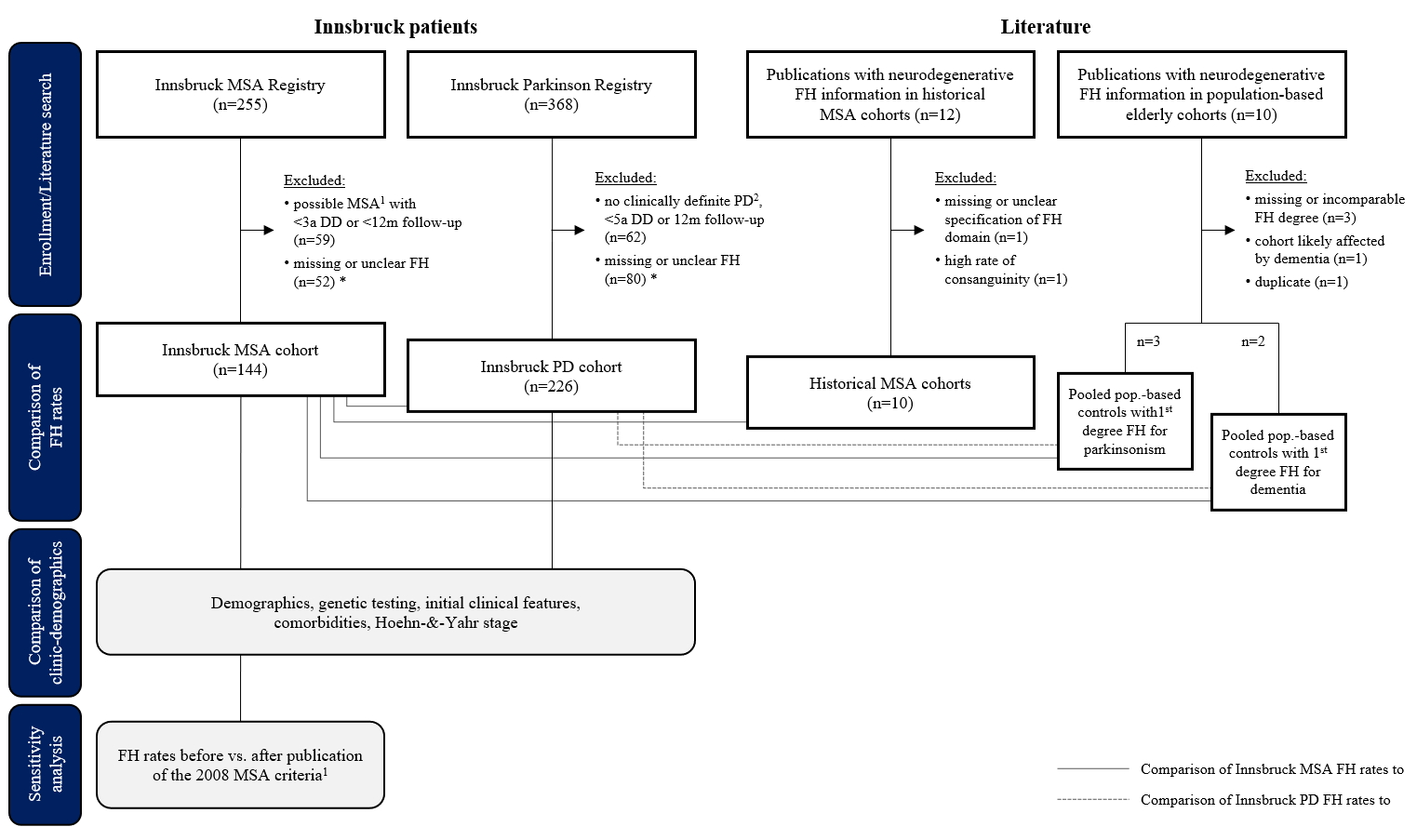


* Included MSA and PD cases did not significantly differ from cases excluded due to missing or unclear FH (n=52 MSA; n=80 PD).

DD, disease duration; FH, family history; MSA, multiple system atrophy; PD, Parkinson‘s disease.

**References**

1. Gilman S, Wenning GK, Low PA, et al. Second consensus statement on the diagnosis of multiple system atrophy. Neurology 2008;71(9):670-676.

2. Lees AJ, Hardy J, Revesz T. Parkinson's disease. Lancet 2009;373(9680):2055-2066.

3. Goetz CG, Poewe W, Rascol O, et al. Movement Disorder Society Task Force report on the Hoehn and Yahr staging scale: status and recommendations. Mov Disord 2004;19(9):1020-1028.

4. Seematter-Bagnoud L, Santos-Eggimann B. Population-based cohorts of the 50s and over: a summary of worldwide previous and ongoing studies for research on health in ageing. European journal of ageing 2006;3(1):41.

5. Agresti A, Coull BA. Approximate Is Better than "Exact" for Interval Estimation of Binomial Proportions. The American Statistician 1998;52(2):119-126.

6. Stankovic I, Quinn N, Vignatelli L, et al. A critique of the second consensus criteria for multiple system atrophy. Mov Disord 2019;34(7):975-984.

7. Obeso JA, Stamelou M, Goetz CG, et al. Past, present, and future of Parkinson's disease: A special essay on the 200th Anniversary of the Shaking Palsy. Mov Disord 2017;32(9):1264-1310.

8. Quadri M, Mandemakers W, Grochowska MM, et al. LRP10 genetic variants in familial Parkinson's disease and dementia with Lewy bodies: a genome-wide linkage and sequencing study. Lancet Neurol 2018;17(7):597-608.

9. Wenning GK, Geser F, Krismer F, et al. The natural history of multiple system atrophy: a prospective European cohort study. Lancet Neurol 2013;12(3):264-274.

10. Wenning GK, Tison F, Seppi K, et al. Development and validation of the Unified Multiple System Atrophy Rating Scale (UMSARS). Mov Disord 2004;19(12):1391-1402.
