# Supplementary material for: Family History for Neurodegeneration in Multiple System Atrophy: Does it Indicate Susceptibility?
Source: Mov Disord. 2022 Aug 27;37(11):2310–2. doi: 10.1002/mds.29202 (PMC9804463; doi:10.1002/mds.29202)
Supplement: Supplementary file 1 — Appendix S1. Supporting Information. [file MDS-37-2310-s001.zip › MDS_29202_Suppl Doc 2_Letter_Family history for neurodegeneration in MSA.docx]

**Family history for neurodegeneration in multiple system atrophy:
does it indicate susceptibility?
A clinico-genetic study based on the Innsbruck MSA Registry**

**Supporting Data**

**Supplementary Table 2.** FH domain and degree adjusted frequency rates of the Innsbruck MSA cases versus historical MSA cohorts

| **Historical publication** | **FH for** | **Degree** | **Methodology** | **Cases** | **Controls** | **Innsbruck MSA** | ***P* vs. historical MSA** | ***P* vs. historical controls** |
| --- | --- | --- | --- | --- | --- | --- | --- | --- |
| *Observational* |  |  |  |  |  |  |  |  |
| Nee *et al.* 1991^1^ | NDD | 1^st^ | Case-control tertiary single center study, American.* | 9 (27.3) | 1 (1.3) | 29 (24.8) | 0.771 | **<0.001** |
| Vanacore *et al.* 2005^2^ | NDD | 1^st^ | Case-control tertiary multicenter study, European.^†^ | 8 (11.0) | 6 (8.2) | 29 (24.8) | **0.020** | **0.004** |
| Wullner *et al.* 2007^3^ | P, T & A | 1^st^ | Cross-sectional tertiary multicenter study, German.^†^ | 21 (9.5) | - | 23 (17.0) | **0.037** | - |
| Vidal *et al.* 2010^4^ | P | 1^st^ | Case-control tertiary multicenter study, French.^†^ | 13 (18.3) | 4 (5.6) | 15 (10.4) | 0.106 | 0.246 |
|  | D |  |  | 11 (15.5) | 10 (14.1) | 5 (4.3) | **0.008** | **0.017** |
| *Retrospective* |  |  |  |  |  |  |  |  |
| Wenning *et al.* 1993^5^ | NDD | 1^st^-3^rd^ | UKPDS brain bank, United Kingdom. | 5 (13.2) | - | 45 (39.5) | **0.003** | - |
| Soma *et al.* 2006^6^ | NDD | 1^st^-2^nd^ | Tertiary single center study, Japanese.^†^ | 3 (1.9) | - | 43 (37.7) | **<0.001** | - |
| Fujioka *et al.* 2014^7^ | P, D & A | 1^st^-3^rd^ | Review, American.^‡^ | 12 (19.7) | - | 37 (32.5) | 0.073 | - |
| Kim *et al.* 2014^8^ | P | 1^st^-3^rd§^ | Tertiary single center study, Korean.^‡^ | 3 (1.1) | - | 26 (18.3) | **<0.001** | - |
|  | A |  |  | 5 (1.8) | - | 4 (2.9) | 0.457 | - |
| Koga *et al.* 2015^9^ | P | 1^st^-3^rd§^ | Mayo clinic brain bank, American. | 9 (11.4) | - | 26 (18.3) | 0.179 | - |
|  | D |  |  | 10 (12.7) | - | 12 (10.3) | 0.604 | - |
| Miki *et al.* 2019^10^ | P & A | 1^st^-2^nd^ | Queen Square brain bank, United Kingdom. | 17 (10.6) | - | 28 (20.6) | **0.017** | - |

Figures are n (%). Bold font indicates statistically significant differences. Two studies were excluded due to missing or unclear information regarding the assessed FH domain and degree,^11^ or high prevalence of consanguinity (20% of the whole study cohort).^12^ Except for Nee et al.^1^, abovementioned studies primarily adopted the *FH method*.^13^

Asterisks indicates MSA diagnosis according to Polinsky 1984.^14^

^†^ indicates MSA diagnosis based on 1^st^ consensus criteria.^15^

^‡^ indicates MSA diagnosis based on 2^nd^ consensus criteria.^16^

^§^ indicates assumed first-to-third degree in absence of detailed specification.

A, ataxia; D, dementia; FH, family history; MSA, multiple system atrophy; NDD, neurodegenerative disorder; P, parkinsonism; T, tremor; UKPDS, United Kingdom Parkinson Disease Society.

**Supplementary Table 3.** Comparison of FH frequency rates and clinic-demographic characteristics between the Innsbruck MSA and PD cohort

|  | **MSA** (n=144) | **PD** (n=226) | ***P*** |
| --- | --- | --- | --- |
| **Demographics** | | | |
| Female patients | 73 (50.7) | 82 (36.3) | **0.006** |
| Age at onset, years | 58.1 [50.6; 63.7] | 60.0 [51.5; 65.8] | 0.056 |
| Age at baseline visit, years | 60.8 [54.3; 67.3] | 64.6 [57.7; 69.7] | **0.004** |
| Disease duration at baseline visit, months | 35.5 [18.6; 49.0] | 25.1 [12.0; 85.5] | 0.043 |
| Age at final visit, years | 63.0 [56.7; 70.6] | 75.5 [69.0; 80.0] | **<0.001** |
| Disease duration at final visit, months | 59.4 [46.8; 78.4] | 169.0 [125.9; 236.0] | **<0.001** |
| Follow-up time, months | 20.7 [4.4; 38.1] | 123.7 [91.2; 175.6] | **<0.001** |
| **FH for neurodegenerative disorders** | | | |
| Neurodegenerative disorders, any degree (n=273) | 45 (39.5) | 85 (53.5) | 0.023 |
| 1^st^ degree  (n=278) | 29 (24.8) | 66 (41.0) | **0.005**^†^ |
| 2^nd^ degree  (n=275) | 23 (20.2) | 28 (17.4) | 0.558 |
| 3^rd^ degree  (n=276) | 5 (4.3) | 8 (5.0) | 1.000 |
| Parkinsonism, any degree (n=357) | 26 (18.3) | 55 (25.6) | 0.108 |
| 1^st^ degree  (n=361) | 15 (10.4) | 37 (17.1) | 0.079 |
| 2^nd^ degree  (n=361) | 12 (8.5) | 20 (9.1) | 0.824 |
| 3^rd^ degree  (n=362) | 3 (2.1) | 7 (3.2) | 0.746 |
| Dementia, any degree (n=278) | 12 (10.3) | 9 (5.6) | 0.136 |
| 1^st^ degree  (n=280) | 5 (4.3) | 9 (5.5) | 0.784 |
| 2^nd^ degree  (n=278) | 7 (6.0) | 0 (0) | **0.002** |
| 3^rd^ degree  (n=278) | 2 (1.7) | 0 (0) | 0.173 |
| Tremor, any degree (n=341) | 10 (7.3) | 28 (13.7) | 0.064 |
| 1^st^ degree  (n=343) | 4 (2.9) | 25 (12.2) | **0.002**^†^ |
| 2^nd^ degree  (n=341) | 5 (3.6) | 7 (3.4) | 1.000 |
| 3^rd^ degree  (n=341) | 1 (0.7) | 1 (0.5) | 1.000 |
| Ataxia, any degree (n=330) | 4 (2.9) | 0 (0) | 0.029 |
| 1^st^ degree  (n=333) | 4 (2.9) | 0 (0) | 0.030 |
| 2^nd^ degree  (n=330) | 0 (0) | 0 (0) | - |
| 3^rd^ degree  (n=331) | 0 (0) | 0 (0) | - |
| Motor-neuron disease, any degree (n=278) | 1 (0.9) | 1 (0.6) | 1.000 |
| 1^st^ degree  (n=280) | 1 (0.9) | 0 (0) | 0.418 |
| 2^nd^ degree  (n=278) | 1 (0.9) | 1 (0.6) | 1.000 |
| 3^rd^ degree  (n=278) | 0 (0) | 0 (0) | - |
| Familial clustering (n=278) | 11 (9.5) | 28 (17.3) | 0.065 |
| **Genetic testing**^‡^ | | | |
| Positive genetic test (n=36) | 0 (0) | 5 (71.4) | **<0.001** |
| **Initial clinical feature** | | | |
| Movement disorder | 122 (84.7) | 220 (97.3) | **<0.001** |
| Parkinsonism | 75 (52.1) | 220 (97.3) | **<0.001** |
| Cerebellar signs | 54 (37.5) | - | * |
| Autonomic failure (n=358) | 34 (24.1) | 7 (3.2) | **<0.001** |
| Orthostatic intolerance  (n=364) | 13 (9.0) | 5 (2.3) | **0.005** |
| Urinary failure  (n=363) | 25 (17.7) | 2 (0.9) | **<0.001** |
| Movement disorder & autonomic failure (n=358) | 12 (8.5) | 1 (0.5) | **<0.001** |
| **Comorbidities** | | | |
| *At baseline visit* |  |  |  |
| Cardiovascular diseases | 48 (33.3) | 77 (34.1) | 0.884 |
| Diabetes mellitus  (n=367) | 11 (7.7) | 12 (5.3) | 0.353 |
| *At final visit* |  |  |  |
| Cardiovascular diseases | 56 (38.9) | 117 (51.8) | **0.015** |
| Diabetes mellitus  (n=365) | 12 (8.5) | 22 (9.9) | 0.650 |
| **Rating scales** | | | |
| *At baseline visit* |  |  |  |
| UMSARS Part IV score  (n=107) | 2.0 [2.0; 3.0] | - | * |
| Hoehn-&-Yahr stage  (n=343) | 3.0 [2.0; 3.0] | 2.0 [1.0; 2.0] | **<0.001** |
| *At final visit* |  |  |  |
| UMSARS Part IV score | 4.0 [3.0; 4.0] | - | * |
| Hoehn-&-Yahr stage  (n=360) | 4.0 [3.0; 5.0] | 3.0 [2.0; 4.0] | **<0.001** |
| **Certainty of MSA diagnosis and predominant phenotype at final visit** | | | |
| Diagnostic certainty, probable^16^ | 127 (88) | - | * |
| Predominant phenotype, parkinsonian | 87 (60) | - | * |

Figures are n (%) or median [25^th^; 75^th^ percentile]. Bold font indicates statistically significant differences after Benjamini-Hochberg correction.

Asterisks indicates not analyzed as not applicable for PD.

^†^ Undetected monogenic forms of parkinsonian or tremor disorders may explain the significantly higher first-degree FH frequency rates for neurodegenerative disorders overall and tremor in the PD cohort.

^‡^ Genetic testing was mainly conducted in cerebellar variant MSA (n=24/29; 83%), with pathogenic mutations in the spinocerebellar ataxia genes (*SCA 1, 2, 3, 6, 17*) tested most often. Genes tested in the PD cohort included *ATP13A2*, *DJ1*, *GBA*, *LRRK2*, *PINK1*, *PRKN*, *SNCA*, and *VPS35,* with positive results for *PRKN* (n=2); *GBA* (n=2); *VPS35* (n=1). The results of the statistical analysis did not change when excluding PD cases with detected genetic mutations.

FH, family history; MSA, multiple system atrophy; PD, Parkinson’s disease; UMSARS, Unified MSA Rating Scale.

**Supplementary Table 4.** Comparison of first-degree FH for parkinsonism and dementia among people with MSA, PD and population-based controls

| **Historical publication** | **Cohort characteristics** | **Age** | **Cases** | **MSA** | ***P* vs. MSA** | **PD** | ***P* vs. PD** |
| --- | --- | --- | --- | --- | --- | --- | --- |
| *FH for 1^st^ degree parkinsonism* | | | | | | | |
| Berg *et al.* 2011^17^ | German, Austrian/South Tyrolean (PRIPS); n=1,847 | >50 | 169 (9.2) |  |  |  |  |
| Darweesh *et al.* 2016^18^ | Dutch (Rotterdam); n=6,492 | >55 | 311 (4.8) |  |  |  |  |
| Giagkou *et al.* 2020^19^ | Greek (Hellenic); n=959 | >65 | 38 (4.0) |  |  |  |  |
| Pooled | n=9,298 | | 518 [5.6 (5.1-6.1)] | 15 [10.4 (6.3-16.6)] | **0.012** | 37 [17.1 (12.6-22.7)] | **<0.001** |
| *FH for 1^st^ degree dementia* | | | | | | | |
| van Ojen *et al.* 1995^20^ | Dutch (AMSTEL); n=3,813 | >65 | 638 (16.7) |  |  |  |  |
| Ott *et al.* 2004^21^ | Dutch (Rotterdam), Danish (Odense), French (Paquid); n=7,673 | >65 | 1,479 (19.3) |  |  |  |  |
| Pooled | n=11,486 | | 2,117 [18.4 (17.7-19.2)] | 5 [4.3 (1.6-9.9)] | **<0.001** | 9 [5.5 (2.8-10.3)] | **<0.001** |

Figures are n (%), or n [% (95%c.i.)] for pooled figures. Bold font indicates statistically significant differences. Five studies were not considered [incomplete information or incomparable FH degree (n=3)^22-24^; cohort likely affected itself by dementia (n=1)^25^; duplicate (n=1)^26^].

AMSTEL, Amsterdam Study of the Elderly; FH, family history; MSA, multiple system atrophy; PD, Parkinson’s disease; PRIPS, Prospective Validation of Risk Factors for the Development of Parkinsonian Syndromes.

**Post-hoc sensitivity analysis on positive FH frequency rates before and after publication of the 2008 MSA criteria**

Two of twelve (17%) MSA cases seen exclusively before August 2008 had a positive FH for a neurodegenerative disorder, 43 of 102 (42%) MSA cases after the publication of the 2008 MSA criteria^16^ (*P*=0.121).

**References**

1. Nee LE, Gomez MR, Dambrosia J, Bale S, Eldridge R, Polinsky RJ. Environmental-occupational risk factors and familial associations in multiple system atrophy: a preliminary investigation. Clin Auton Res 1991;1(1):9-13.

2. Vanacore N, Bonifati V, Fabbrini G, et al. Case-control study of multiple system atrophy. Mov Disord 2005;20(2):158-163.

3. Wüllner U, Schmitz-Hübsch T, Abele M, Antony G, Bauer P, Eggert K. Features of probable multiple system atrophy patients identified among 4770 patients with parkinsonism enrolled in the multicentre registry of the German Competence Network on Parkinson's disease. J Neural Transm (Vienna) 2007;114(9):1161-1165.

4. Vidal JS, Vidailhet M, Derkinderen P, Tzourio C, Alpérovitch A. Familial aggregation in atypical Parkinson's disease: a case control study in multiple system atrophy and progressive supranuclear palsy. J Neurol 2010;257(8):1388-1393.

5. Wenning GK, Wagner S, Daniel S, Quinn NP. Multiple system atrophy: sporadic or familial? Lancet 1993;342(8872):681.

6. Soma H, Yabe I, Takei A, Fujiki N, Yanagihara T, Sasaki H. Heredity in multiple system atrophy. J Neurol Sci 2006;240(1-2):107-110.

7. Fujioka S, Ogaki K, Tacik PM, Uitti RJ, Ross OA, Wszolek ZK. Update on novel familial forms of Parkinson's disease and multiple system atrophy. Parkinsonism Relat Disord 2014;20 Suppl 1(0 1):S29-34.

8. Kim HJ, Jeon BS, Shin J, et al. Should genetic testing for SCAs be included in the diagnostic workup for MSA? Neurology 2014;83(19):1733-1738.

9. Koga S, Aoki N, Uitti RJ, et al. When DLB, PD, and PSP masquerade as MSA: an autopsy study of 134 patients. Neurology 2015;85(5):404-412.

10. Miki Y, Foti SC, Asi YT, et al. Improving diagnostic accuracy of multiple system atrophy: a clinicopathological study. Brain 2019;142(9):2813-2827.

11. Chen YP, Zhao B, Cao B, et al. Mutation scanning of the COQ2 gene in ethnic Chinese patients with multiple-system atrophy. Neurobiol Aging 2015;36(2):1222.e1227-1211.

12. Nasri A, Ben Djebara M, Sghaier I, et al. Atypical parkinsonian syndromes in a North African tertiary referral center. Brain and behavior 2021;11(1):e01924.

13. Khoury MJ, Beaty TH, Beaty TH, Cohen BH. Fundamentals of genetic epidemiology: Monographs in Epidemiology and, 1993.

14. Polinsky RJ. Multiple system atrophy. Clinical aspects, pathophysiology, and treatment. Neurologic clinics 1984;2(3):487-498.

15. Gilman S, Low PA, Quinn N, et al. Consensus statement on the diagnosis of multiple system atrophy. J Neurol Sci 1999;163(1):94-98.

16. Gilman S, Wenning GK, Low PA, et al. Second consensus statement on the diagnosis of multiple system atrophy. Neurology 2008;71(9):670-676.

17. Berg D, Seppi K, Behnke S, et al. Enlarged substantia nigra hyperechogenicity and risk for Parkinson disease: a 37-month 3-center study of 1847 older persons. Arch Neurol 2011;68(7):932-937.

18. Darweesh SK, Koudstaal PJ, Stricker BH, Hofman A, Steyerberg EW, Ikram MA. Predicting Parkinson disease in the community using a nonmotor risk score. European journal of epidemiology 2016;31(7):679-684.

19. Giagkou N, Maraki MI, Yannakoulia M, et al. A Prospective Validation of the Updated Movement Disorders Society Research Criteria for Prodromal Parkinson's Disease. Mov Disord 2020;35(10):1802-1809.

20. van Ojen R, Hooijer C, Bezemer D, Jonker C, Lindeboom J, van Tilburg W. Late-life depressive disorder in the community. II. The relationship between psychiatric history, MMSE and family history. The British journal of psychiatry : the journal of mental science 1995;166(3):316-319.

21. Ott A, Andersen K, Dewey ME, et al. Effect of smoking on global cognitive function in nondemented elderly. Neurology 2004;62(6):920-924.

22. Qian J, Wolters FJ, Beiser A, et al. APOE-related risk of mild cognitive impairment and dementia for prevention trials: An analysis of four cohorts. PLoS medicine 2017;14(3):e1002254.

23. Noale M, Limongi F, Zambon S, Crepaldi G, Maggi S. Incidence of dementia: evidence for an effect modification by gender. The ILSA Study. International psychogeriatrics 2013;25(11):1867-1876.

24. Lindsay J, Laurin D, Verreault R, et al. Risk factors for Alzheimer's disease: a prospective analysis from the Canadian Study of Health and Aging. Am J Epidemiol 2002;156(5):445-453.

25. Matthews FE, Chatfield M, Brayne C. An investigation of whether factors associated with short-term attrition change or persist over ten years: data from the Medical Research Council Cognitive Function and Ageing Study (MRC CFAS). BMC public health 2006;6:185.

26. Fahim S, van Duijn CM, Baker FM, et al. A study of familial aggregation of depression, dementia and Parkinson's disease. European journal of epidemiology 1998;14(3):233-238.
